# Prevalence, incidence and healthcare burden of eosinophilic granulomatosis with polyangiitis in the UK

**DOI:** 10.1183/23120541.00430-2023

**Published:** 2024-05-13

**Authors:** Jeremiah Hwee, Lorraine Harper, Qinggong Fu, Krishnarajah Nirantharakumar, George Mu, Rupert W. Jakes

**Affiliations:** 1 GSK, Epidemiology, Mississauga, ON, Canada; 2 University of Birmingham, Institute of Applied Health Research, Birmingham, UK; 3 GSK, Value Evidence Outcomes, Collegeville, PA, USA; 4 GSK, Epidemiology, London, UK

## Abstract

**Background:**

Eosinophilic granulomatosis with polyangiitis (EGPA) is a rare but serious disease characterised by the combination of small-to-medium vessel vasculitis, blood and tissue eosinophilia, and asthma and/or sinonasal disease. This study estimated the prevalence and incidence of diagnosed EGPA in the United Kingdom (UK), and described the demographics, clinical characteristics and healthcare resource utilisation (HCRU) of this population.

**Methods:**

This retrospective longitudinal study of patients with newly diagnosed EGPA (index) (2005–2019) used the Clinical Practice Research Datalink AURUM and Hospital Episode Statistics databases. The primary outcomes were the annual prevalence (2005–2019) and incidence (2006–2019) of EGPA, and secondary outcomes included patient demographics and clinical characteristics, and HCRU in the year pre- and post-index (diagnosis).

**Results:**

Populations of patients with EGPA comprised 940 prevalent cases and 502 incident cases, of which 377 were linked to Hospital Episode Statistics. EGPA prevalence increased from 22.7 to 45.6 cases per 1 000 000 (2005–2019), driven by patients aged ≥18 years. Incidence ranged from 2.3 to 4.0 per 1 000 000 person-years (2006–2019). Pre-index, the most common clinical symptoms were respiratory related, and the most common comorbidities were asthma (80.6%) and nasal polyps (32.1%). Post-index, 19.1% had an EGPA-related inpatient stay (median length of stay 11.0 days) and 38.7% had five or more oral corticosteroid (OCS) prescriptions with a mean OCS possession ratio per patient of 47.0%.

**Conclusions:**

Although EGPA incidence in the UK remains relatively stable, prevalence is increasing, and HCRU and OCS use remain frequent, suggesting considerable healthcare burden for patients with EGPA.

## Tweetable abstract

**EGPA increased in prevalence from 2005 to 2019 in the UK; incidence remained stable. Post-diagnosis, 19% of patients had EGPA-related inpatient stays and 80% required oral corticosteroids, highlighting the high healthcare burden and severity of EGPA.** https://bit.ly/3NOHoln

## Introduction

Eosinophilic granulomatosis with polyangiitis (EGPA) is a rare disease characterised by eosinophilic inflammation and necrotising vasculitis of small/medium-sized blood vessels [1–3]. EGPA is a type of antineutrophil cytoplasmic antibody (ANCA)-associated vasculitis (AAV) [2, 3], although ANCAs are only detected in ∼30–40% of cases [4, 5]. Elevated eosinophil counts in the blood and tissue, vasculitis and granuloma formation are all thought to contribute towards multiple organ injury and impairment [6]. EGPA is commonly characterised by asthma, elevated eosinophil counts, neuropathy and sinusitis [2, 3, 5, 7].

EGPA treatment, typically oral corticosteroids (OCS) and immunosuppressants, aims to induce remission and reduce disease relapses [8, 9]. However, OCS and immunosuppressants are associated with significant toxicity, particularly with chronic exposure [10, 11]. Additionally, not all patients achieve remission and others may experience exacerbations or relapses, especially when treatments are tapered [5, 12, 13], which, together with the wide range of organ systems involved, necessitates frequent healthcare resource utilisation (HCRU) [5, 9, 14, 15].

The prevalence and incidence of EGPA varies globally, with estimated prevalence of 2.0–38.0 per 1 000 000 people [14–17], and incidence of 1.2 per 1 000 000 person-years [14]. EGPA is a rare and challenging diagnosis, which is often misdiagnosed [18]. Therefore, local differences in awareness and recognition among healthcare providers may contribute to regional variations, as has been observed in other AAVs [18, 19]. Another contributing factor may be the changing classification criteria over time [2, 3, 20, 21]. Given the rarity of and difficulty diagnosing EGPA, limited information is available on the prevalence, incidence and associated burden of disease in the United Kingdom (UK) [17, 22].

This study aimed to estimate the prevalence and incidence of diagnosed EGPA in the UK, and to describe the demographics, clinical characteristics and HCRU of patients following EGPA diagnosis.

## Materials and methods

### Study design and data sources

**FIGURE 1:**
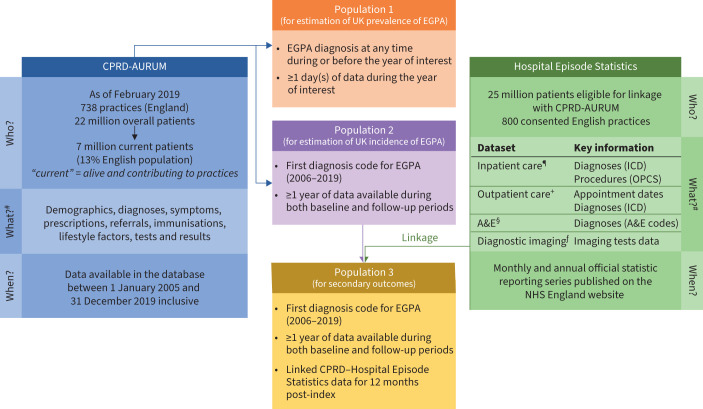
Data sources and analysis populations. CPRD: Clinical Practice Research Datalink; EGPA: eosinophilic granulomatosis with polyangiitis; ICD: International Classification of Diseases; OPCS: Office of Population Censuses and Surveys's Classification of Surgical Operations and Procedures; A&E: accident and emergency; NHS: National Health Service. ^#^: information from [24]; ^¶^: from 1997; ^+^: from 2003; ^§^: from 2007; ^ƒ^: from 2012.

This was a retrospective, longitudinal study of patients newly diagnosed with EGPA (1 January 2005 to 31 December 2019) using the Clinical Practice Research Datalink (CPRD)-AURUM and Hospital Episode Statistics databases [23, 24]. The index date was the date of the first EGPA diagnosis during the study period based on the presence of a medcode identifier, Read code, Education Management Information System (EMIS) code or SNOMED identifier code for EGPA, allergic granulomatous angiitis or Churg-Strauss syndrome (supplementary table S1). The baseline and follow-up periods included the year pre- and post-index, respectively (supplementary figure S1). The CPRD-AURUM database (figure 1) consists of anonymised, longitudinal medical records of patients registered with contributing primary care practices across the UK (predominantly England and Northern Ireland) and contains data collected routinely from participating practices using the EMIS Web electronic patient record system software, including data on demographics, lifestyle factors, diagnoses, symptoms, prescriptions, referrals and medical tests [24]. As of February 2019, CPRD-AURUM contained data of >22 million patients from 738 general practitioner (GP) practices in England, of whom 7 million were active (still alive and registered with a GP practice), representing a coverage of ∼13% of the population of England. The CPRD-AURUM resource was launched in 2017, but the database includes a full historic collection of the coded part of each practice's electronic health records. Further characterisation of the data source has been published previously [24].

Anonymised data from CPRD-AURUM can be individually linked to secondary care and other health and area-based datasets, including the Hospital Episode Statistics database (figure 1). Linkage of CPRD-AURUM with Hospital Episode Statistics is possible for a subset of ∼25 million patients currently registered with 800 consented English practices that actively participate in the linkage scheme. The Hospital Episode Statistics database contains details of all inpatient episodes of care, outpatient appointments, and accident and emergency (A&E) attendances and diagnostic imaging at National Health Service (NHS) hospitals in England. These data are collected primarily for administrative purposes, although they are designed to enable secondary use. The inpatient data (Hospital Episode Statistics – Admitted Patient Care) includes coded diagnoses (using the International Classification of Diseases, tenth revision (ICD-10) codes), operations and procedures (Office of Population Censuses and Surveys, fourth revision (OPCS 4) codes), as well as patient demographic, admission and discharge information. Outpatient data contain appointment dates and times, and specialties, but limited clinical information [24].

Informed consent and ethics committee or institutional review board approval were not required as no direct patient contact or primary collection of patient data occurred. The CPRD obtains ethical research approval annually from the UK's Health Research Authority Research Ethics Committee to accumulate and distribute patient data.

### Patient eligibility

Three patient populations were defined (figure 1). Populations 1 and 2 were based on CPRD-AURUM data only. To calculate the annual prevalence of EGPA (2005–2019) in the UK, population 1 was defined as patients with a diagnosis for EGPA at any time during or before the year of interest, ≥1 day(s) of CPRD-AURUM data during the year of interest. To calculate the incidence of EGPA in the UK, population 2 was defined as patients with a first diagnosis code for EGPA (2006–2019) and ≥1 calendar year of CPRD-AURUM data during both the baseline and follow-up periods. For the secondary outcomes, population 3 was defined as patients with a first diagnosis for EGPA (2006–2019) with at least one calendar year of CPRD-AURUM data records during baseline and follow-up periods, and linked to Hospital Episode Statistics data for 12 months post-index. To ensure only incident cases were captured accurately in populations 2 and 3, only patients with no diagnosis of EGPA during baseline were included.

### Study outcomes

The primary outcome was the annual prevalence of diagnosed EGPA (population 1, overall, and stratified by age (0–17 and ≥18 years)) in 2005–2019 and the annual incidence rate of EGPA diagnosis in 2006–2019 (population 2).

Secondary outcomes included demographics at index, and clinical characteristics and Charlson Comorbidity Index (CCI) score during baseline, and HCRU (including OCS use) during the follow-up period (population 3). As Hospital Episode Statistics data are specific for England, the secondary outcomes data reflect an English rather than UK population. The CPRD-AURUM and Hospital Episode Statistics databases were used to identify clinical symptoms and comorbidity conditions using codes from a previous study (available upon request) [25].

### Statistical analysis

This was a descriptive study, and neither hypothesis tests were conducted, nor formal power calculation performed. However, a feasibility assessment was performed, including widths calculations of the 95% confidence intervals for prevalence and incidence estimations, detailed in the supplementary methods and supplementary table S2.

EGPA prevalence was calculated as the number of patients with an EGPA diagnosis during and before a particular calendar year, divided by the number of patients with a calendar year of data in the CPRD-AURUM database on 31 December in each calendar year. EGPA incidence was calculated as the number of patients with an incident EGPA diagnosis from 1 January to 31 December in the calendar year of interest, divided by the total number of days at risk. For incidence rate, patients had at least 365 days after first registration in CPRD-AURUM prior to contributing to time at risk between 2006 and 2019. Time at risk started on day 366 after registration. This was to ensure that the incident cases were accurate and not an existing diagnosis that was recorded at time of registration.

All secondary outcomes were also analysed descriptively using mean±sd or median and interquartile range (IQR) for continuous variables and frequency (%) for categorical variables. CCI score was calculated using the Metcalfe adaptation [26]. HCRU assessments included the proportion of patients with at least one event and mean number of events including EGPA-related and all-cause inpatient stays, all-cause A&E visits, specialist outpatient visits, all-cause outpatient visits, all-cause procedures and all-cause primary care visits. For inpatient stays, the cumulative and median length of stays was also reported. OCS use was measured according to the number of prescriptions throughout the year and split into quartiles, total prescriptions, and average days per year of use. The OCS medication possession ratio (MPR) was calculated based on the total number of days covered by OCS prescriptions (derived using quantity/daily dose variables) during the follow-up period divided by duration.

## Results

### Patient populations

Population 1 and population 2 included 940 prevalent patients and 502 incident patients, respectively. There were 377 patients aged ≥18 years who were successfully linked to CPRD–Hospital Episode Statistics and eligible for inclusion in population 3.

### Demographics and clinical characteristics

Patient demographics and clinical characteristics are shown in table 1. The mean±sd age at index was 57.4±14.2 years among 377 patients aged ≥18 years: 2% of patients were aged 18–25 years, 66% were aged 26–64 years and 32% were aged ≥65 years. Additionally, fewer than five patients were aged ≤17 years and were not included in population 3 for the secondary outcomes (for clinical characteristics/conditions with fewer than five patients, CPRD required data to be suppressed to minimise the risk of patient identification). In total, 51.2% of patients were female, and 84.6% had a CCI score ≥1. Blood eosinophil counts (BECs) at diagnosis were elevated, with a geometric mean±sd (95% CI) BEC of 1385.5±4.3 (1163.4–1649.9) cells·μL^−1^ (normal range 50–500 cells·μL^−1^) [27]. Only 13.8% of patients had BECs <400 cells·μL^−1^, while 38.2% had BECs ≥1000 cells·μL^−1^ (table 1). The most common clinical symptoms during baseline were cough/breathlessness (37.7%) and ear, nose and throat involvement (18.8%). The most common comorbidities pre-index were asthma (80.6%) and nasal polyps (32.1%).

**TABLE 1:** Patient demographics^#^ at index and clinical characteristics during the baseline period^¶^

**Patients**	377
**Age at index, years**	
Mean±sd	57.4±14.2
Median (IQR)	58 (48–68)
≤17	^ƒ^
18–25	6 (1.6)
26–64	249 (66.1)
≥65	122 (32.4)
**Females at index**	193 (51.2)
**CCI score during baseline**	
0	58 (15.4)
1	226 (60.0)
2	56 (14.9)
≥3	37 (9.8)
**Blood eosinophil count during baseline^+^, cells·µL^−1^**	
Median (IQR)	1170 (500–4800)
<400	52 (13.8)
≥400–<1000	76 (20.2)
≥1000	144 (38.2)
Missing	105 (27.9)
**Clinical symptoms during baseline** **^§^**	
Cough or breathlessness	142 (37.7)
ENT involvement	71 (18.8)
Nonspecific chest symptoms	37 (9.8)
Skin involvement	30 (8.0)
Constitutional manifestations	28 (7.4)
Musculoskeletal involvement	15 (4.0)
Renal involvement	16 (4.2)
Gastrointestinal involvement	21 (5.6)
Eye involvement	7 (1.9)
Chest pain	<5^##^
**Comorbid conditions at any time prior to index** **^§^**	
Asthma	304 (80.6)
Nasal polyposis	121 (32.1)
Chronic rhinosinusitis	91 (24.1)
Allergic rhinitis	61 (16.2)
Peripheral neuropathy	43 (11.4)
Ischaemic stroke	16 (4.2)
COPD	15 (4.0)
Cardiomyopathy	9 (2.4)
Hypereosinophilic syndrome	<5^##^
Heart failure	<5^##^

Data are presented as n or n (%), unless otherwise stated. IQR: interquartile range; CCI: Charlson Comorbidity Index; ENT: ear, nose and throat. ^#^: patients from population 3; ^¶^: baseline period defined as the year before index (inclusive); ^+^: the maximum value was reported if multiple values were available; ^§^: ≥1 code for characteristic of interest; ^ƒ^: patients aged 0–17 years were not included due to the small number (fewer than five) of patients included in this age group; ^##^: for clinical characteristics/conditions with fewer than five patients, Clinical Practice Research Datalink required data to be suppressed to minimise the risk of patient identification.

### Prevalence and incidence of EGPA

**FIGURE 2:**
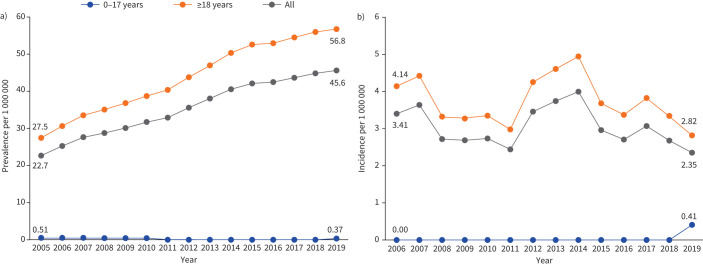
a) Prevalence and b) incidence of eosinophilic granulomatosis with polyangiitis in the United Kingdom over time*.* Patients from population 1 (940 prevalent patients) and population 2 (502 incident patients).

The overall annual prevalence of diagnosed EGPA increased from 22.7 (95% CI 20.0–25.7) to 45.6 (95% CI 42.1–49.4) cases per 1 000 000 people from 2005 to 2019 (figure 2a and supplementary table S3). The increase was driven by increased prevalence in patients aged ≥18 years. The prevalence in the paediatric population aged ≤17 years ranged between 0 and 0.51 (95% CI 0.01–2.84) per 1 000 000 people over the same period.

Between 2006 and 2019, the overall incidence of EGPA diagnosis ranged between 2.3 (95% CI 1.6–3.4) and 4.0 (95% CI 2.9–5.4) cases per 1 000 000 person-years (figure 2b and supplementary table S3) and the incidence in patients aged ≥18 years ranged between 2.8 (95% CI 1.9–4.1) and 5.0 (95% CI 3.6–6.6) per 1 000 000 person-years. The incidence estimates in patients aged ≤17 years was 0 per 1 000 000 person-years for all years from 2006 to 2018 and 0.41 (95% CI 0.01–2.30) per 1 000 000 person-years in 2019.

### HCRU and OCS use in the 12 months following EGPA diagnosis in England

In the first 12 months following EGPA diagnosis, 49.9% of patients had all-cause inpatient stays and 19.1% had EGPA-related inpatient stays (table 2). The mean±sd number of annual EGPA-related inpatient stays was 1.2±0.6 per patient, with a median (IQR) length of stay of 11 (6.0–17.0) days. 5% of patients required all-cause A&E visits, with a mean annual number of 1.8±1.7 visits per patient. Overall, 97.1% of patients had GP visits and 88.6% had outpatient visits (table 2). The most common specialist outpatient visits were with respiratory medicine (33.7% of patients, with an annual mean of 3.9±2.8 visits per patient), followed by general medicine (32.9% of patients, with an annual mean of 3.5±3.6 visits per patient) and rheumatology (31.8% of patients, with an annual mean of 2.8±2.6 visits per patient). The mean number of GP, nurse or allied health professional visits per patient per year was 16.0±11.1, 3.4±3.9 and 7.2±8.9, respectively.

**TABLE 2:** Healthcare resource utilisation in the year following eosinophilic granulomatosis with polyangiitis (EGPA) diagnosis in England

	**Patients** ^#^	**Events per patient per year**		**Length of stay, days**	
		**Mean±sd**	**Median (IQR)**	**Total**	**Median (IQR)**
**Patients** **^¶^**	377				
**Inpatient stays**					
All-cause	188 (49.9)	1.7±1.3		2992	8.0 (3.0–17.0)
EGPA-related	72 (19.1)	1.2±0.6		1283	11.0 (6.0–17.0)
**All-cause A&E visits**	19 (5.0)	1.8±1.7	1.0 (1.0–2.0)		
**Outpatient visits to specialist** **^+^**					
Respiratory medicine	127 (33.7)	3.9±2.8	3.0 (2.0–5.0)		
General medicine	124 (32.9)	3.5±3.6	2.0 (1.0–4.0)		
Rheumatology	120 (31.8)	2.8±2.6	2.0 (1.0–3.0)		
ENT	95 (25.2)	2.8±1.9	2.0 (1.0–4.0)		
Allied health professional episode	70 (18.6)	2.6±1.9	2.0 (1.0–3.0)		
Ophthalmology	54 (14.3)	2.8±2.0	2.0 (1.0–4.0)		
Nursing episode	48 (12.7)	2.5±2.6	1.0 (1.0–3.0)		
General surgery	47 (12.5)	2.1±2.0	1.0 (1.0–3.0)		
Dermatology	46 (12.2)	2.3±1.7	2.0 (1.0–3.0)		
Nephrology	37 (9.8)	4.2±2.5	4.0 (2.0–6.0)		
**All-cause procedures**	196 (52.0)	6.8±6.2	5.0 (2.0–8.0)		
**All-cause outpatient visits**	334 (88.6)	9.8±7.4	8.0 (4.0–13.0)		
**All-cause primary care visits**					
General practitioner	366 (97.1)	16.0±11.1	14.0 (8.0–22.0)		
Nurse	145 (38.5)	3.4±3.9	2.0 (1.0–4.0)		
Allied health professional	251 (66.6)	7.2±8.9	4.0 (2.0–9.0)		

Data are presented as n or n (%), unless otherwise stated. IQR: interquartile range; A&E: accident and emergency; ENT: ear, nose and throat. ^#^: number of patients with one or more event; **^¶^**: patients from population 3; ^+^: top 10 most frequent specialty outpatient visits.

**FIGURE 3:**
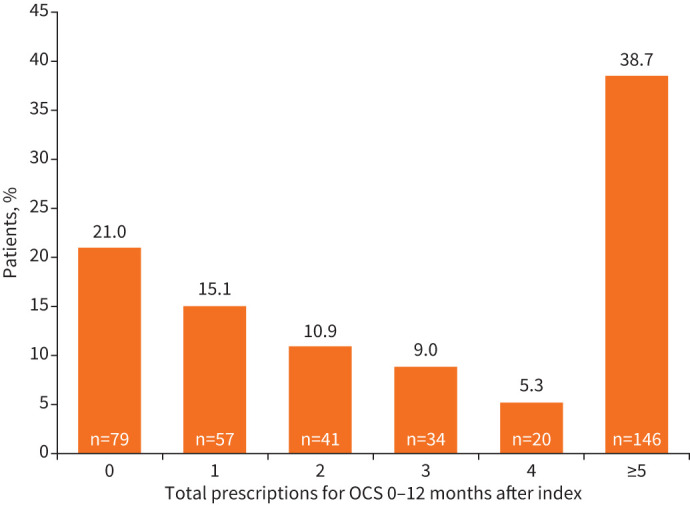
Prescriptions for oral corticosteroids (OCS) in the 12 months after index in England. Patients from population 3.

OCS use was high, with 38.7% of patients having five or more prescriptions for OCS during the 12-month follow-up period (figure 3). The proportion of patients with no OCS prescriptions increased as time from diagnosis lengthened, with 36.3% requiring no OCS 0–3 months post-index, increasing to 55.2% 9–12 months post-index (table 3). Patients had OCS prescriptions covering a mean of 47.0% of days in the year following diagnosis (MPR=0.47).

**TABLE 3:** Oral corticosteroid (OCS) use in the year following eosinophilic granulomatosis with polyangiitis diagnosis in England^#^

**Patients** **^¶^, n**	377
**OCS prescriptions**	
0–≤3 months post-index	
0	137 (36.3)
1	107 (28.4)
2	58 (15.4)
3	52 (13.8)
4	11 (2.9)
≥5	12 (3.2)
>3–≤6 months post-index	
0	178 (47.2)
1	77 (20.4)
2	53 (14.1)
3	40 (10.6)
4	17 (4.5)
≥5	12 (3.2)
>6–≤9 months post-index	
0	201 (53.3)
1	64 (17.0)
2	52 (13.8)
3	33 (8.8)
4	20 (5.3)
≥5	7 (1.9)
>9–≤12 months post-index	
0	208 (55.2)
1	66 (17.5)
2	44 (11.7)
3	36 (9.6)
4	12 (3.2)
≥5	11 (2.9)
**Medication possession ratio, mean±sd**	0.47±0.47

Data are presented as n (%), unless otherwise stated. ^#^: patients from population 3; ^¶^: patients with at least one event.

## Discussion

EGPA is a rare disease and previous estimates of the incidence and prevalence are limited [17, 22, 28, 29]. To our knowledge, this is the first study assessing the prevalence and incidence of diagnosed EGPA exclusively in the UK, together with the associated disease burden. This study reported prevalence and incidence estimates of EGPA in the UK population of 22.7–45.6 per 1 000 000 people and 2.3–4.0 per 1 000 000 person-years, respectively, which is higher than estimates reported for other European countries between 1992 and 2017 [14]. Furthermore, the annual EGPA prevalence increased over the study period, driven by increases in adult prevalence; whereas, overall, the incidence remained stable in all age-ranges. The results presented herein suggest a high healthcare burden for patients with EGPA in the UK, as well as a treatment burden suggested by the high OCS use in this population. This highlights an unmet clinical need that could potentially be addressed by optimised management and/or new optimised treatments. Recent published guidelines have highlighted the use of newer treatments such as biologics, including anti-interleukin (IL)-5 therapies, for the induction of remission or the maintenance of remission for patients with EGPA [18]. Clinical benefits of anti-IL-5 therapies for patients with EGPA include OCS-sparing effects [30–32].

Patient demographics and clinical characteristics were similar to those reported in previous retrospective database studies assessing the prevalence, incidence and burden of EGPA in other countries [5, 9, 15, 33]. Prior to diagnosis patients most commonly experienced respiratory-related symptoms and >80% had comorbid asthma. This is consistent with the commonly reported pattern of disease development leading to EGPA where the development of asthma typically pre-dates the development of hypereosinophilia and vasculitis by several years [5, 7].

Previously reported European and global pooled estimates from a meta-analysis of observational studies covering study periods from 1992 to 2017 indicated an EGPA prevalence of 12.1–15.3 per 1 000 000 and an incidence of 1.1–1.2 per 1 000 000 person-years, although individual studies varied substantially. However, the results of that meta-analysis, and the underlying original studies, have limitations, including the change of criteria for identifying patients with EGPA over time and their inconsistency across studies, and the estimates for prevalence were heavily influenced by the high patient sample of one particular study from claims databases in the United States of America (USA) [14]. In the UK, data from the early 2000s suggested a prevalence of 38.0 per 1 000 000 (2000) and an incidence of 4.2 per 1 000 000 person-years (2004), but with no clear explanation of the methods employed to obtain such estimates [22]. Similarly, a previous England-based study, which analysed Hospital Episode Statistics data, indicated a prevalence of 31.8 cases per 1 000 000 in 2016 [17]. By comparison, in the current study, the 2016 prevalence of EGPA was estimated to be 42.5 cases per 1 000 000. This discrepancy may be due to differences in data source, study methodology and reporting period. For example, the current study includes primary care data from across the UK (CPRD-AURUM database), whereas the England-based study only utilised Hospital Episode Statistics and therefore would not have captured patients seen in primary care but not treated in the hospital setting in that period, which may have not captured less severe cases of EGPA [17]. Finally, the previous study estimated the point prevalence on a given day in 2016, whereas our study estimated annual prevalence from 2005 to 2019.

In the current study, prevalence of EGPA increased two-fold from 2005 to 2019, while EGPA incidence varied, but had no overall increase. Similarly, a retrospective study of administrative claims from the Japan Medical Data Centre (JMDC) claims database (132 patients) found a nine-fold increase in EGPA prevalence from 4.2 to 38.0 per 1 000 000 from 2005 to 2017, where EGPA cases were diagnosed *via* ICD-10 code for EGPA (M30.1), plus an additional ICD-10 code for allergic rhinitis, asthma or chronic sinusitis prior to their EGPA diagnosis [15]. This trend of increasing EGPA prevalence in Japan has continued between 2017 and 2020 [34]. This is consistent with previous studies in Australia and France, which showed two- to three-fold increases in EGPA prevalence over 8–10 years from the late-1990s to mid-2000s, but with little change in incidence, although both studies were small with only eight and 31 EGPA cases identified, respectively [35, 36]. However, the previously mentioned systematic review and meta-analysis study reported no strong trends for increasing EGPA prevalence over time [14]. These apparent differences in prevalence highlight the difficulties in determining accurate prevalence estimates, and may reflect the impact of EGPA rarity, difficulty of diagnosis and disease under-recognition [37]. Nonetheless, EGPA prevalence may have increased over time due to changes to the diagnostic criteria, increased disease awareness and/or the combination of a stable incidence rate and high long-term survival rates [2, 14, 20, 21, 38]. The cumulative survival rate for patients with EGPA at 5 and 10 years from disease onset ranges between 89–97% and 79–89%, respectively [5, 13, 37, 39, 40]. Conventional therapy for EGPA allows for high overall survival rates, and patients with EGPA are living longer, despite living with a high disease burden [9, 41].

HCRU in the year after a EGPA diagnosis was common, with half of patients having an inpatient stay for any reason and almost one-fifth of patients having an EGPA-related inpatient stay. On average, patients had one EGPA-related inpatient visit per year, staying for a median of 11 days per visit. Given the cost of inpatient treatment and the high demand for hospital beds [33], the extended length of hospital stays for EGPA-related treatment demonstrates the sizeable per-patient disease burden for the UK health system. The high discrepancy between all-cause and EGPA-related inpatient stays may reflect an underestimation of the latter due to the challenge in attributing the varied clinical manifestations to EGPA [5, 12], and complications from OCS use [10, 18]. Additionally, many patients with EGPA experience asthma-related inpatients stays [9] and EGPA may therefore not be reported as the primary reason for such stays. The high HCRU burden of EGPA identified in this study is consistent with that demonstrated in other countries [9, 14, 15, 33]. For example, a previous systematic review and meta-analysis, which included studies from the USA, Europe, Australia and Japan, indicated that ∼42% of patients with EGPA required an unscheduled hospital visit [14]. Furthermore, the study found that patients with EGPA required a median of one (range 0–6) hospital visit and one (range 0–12) A&E visit annually [14], consistent with the data reported here.

The high OCS use observed in this study is broadly consistent with the OCS dependence demonstrated in previous studies [9, 15, 33]. Indeed, 38.7% of patients accumulated five or more OCS prescriptions over the year following diagnosis, although there is some evidence that these became less frequent with increasing time from diagnosis. Data on OCS dose were not available here, but previous studies have demonstrated a requirement for high-dose OCS among patients with EGPA. For example, a retrospective Japan-based study found that OCS dose reduced from baseline (mean of 39.1 mg·day^−1^) in the year following an EGPA diagnosis, but remained high in absolute terms (mean of 9.8 mg·day^−1^ and most patients had daily dose ≥15 mg·day^−1^) [15]. Combined with this study's results, these observations suggest that patients with EGPA remain dependent on OCS, increasing the potential for OCS-related toxicity [8, 42].

The burden of acute and chronic corticosteroid-related complications and associated HCRU in severe asthma and the risks increase with cumulative corticosteroid exposure are well documented [11, 42]. Treatment guidelines for EGPA highlight the importance of minimising OCS exposure [8], and novel OCS-sparing therapies that control symptoms while reducing treatment-related side-effects are needed. Given the role of eosinophils in the pathology of EGPA, biologics targeting IL-5, the major cytokine responsible for eosinophil differentiation, survival and activation [43–45], have been investigated for use in EGPA, and have shown benefit as OCS-sparing treatments [30, 46]. The anti-IL-5 monoclonal antibody mepolizumab is approved for the treatment of eosinophil-driven diseases including EGPA in multiple regions worldwide [47–49]; however, anti-IL-5 therapies are not currently approved by the National Institute for Health and Care Excellence in the UK for the treatment of EGPA.

A strength of this study was that it utilised the UK-wide CPRD-AURUM database to assess the prevalence and incidence of EGPA, as well as the England-specific Hospital Episode Statistics database which captures a patient's complete NHS HCRU profile. As of 2019, the CPRD-AURUM database included data from ∼13% of the population in England [24]. In another study with a similar approach, the use of ICD codes in the Hospital Episode Statistics database for the diagnosis of AAV was validated, as these codes were found to have an 86% positive predictive value [17]. Although diagnosis was obtained in CPRD-AURUM *via* different coding systems in the present study, this solidifies the Hospital Episode Statistics database as a promising data source for linkage to CPRD-AURUM for retrospective studies in EGPA. In terms of limitations, reasons for OCS use and the OCS dose were not captured in the Hospital Episode Statistics database, so it was not possible to distinguish whether OCS prescriptions were for EGPA or other comorbid conditions, or to calculate cumulative steroid exposure. EGPA diagnosis can be complicated by the heterogeneous nature of the disease, the need to exclude “vasculitis mimics” and other small/medium-vessel vasculitis, and overlap with other eosinophilic diseases, which can lead to delayed diagnosis or misdiagnosis [1–3]. Consequently, prevalence and incidence could have been underestimated. Furthermore, it is possible that a patient may have had a previous EGPA diagnosis from a non-CPRD-AURUM practice, which could have resulted in previously diagnosed patients being incorrectly included in the first diagnosis/incidence population. Moreover, the number of all-cause hospitalisations being nearly three-fold that of EGPA-related hospitalisations in this study might suggest under-coding. Additionally, the findings of the study may not be generalisable to practices and patients not enrolled in CPRD-AURUM, although a previous assessment of the database found that it was representative of the English population [24]. Finally, this study also shares limitations typical of retrospective database studies, such as potential inconsistencies and errors in the diagnostic codes used to identify EGPA and comorbidities.

### Conclusion

In the UK, although the incidence of EGPA remains relatively stable, the prevalence of EGPA is increasing. This study adds to the currently limited UK-specific data on EGPA prevalence and incidence, and identifies for the first time the considerable healthcare burden for patients with EGPA in the UK, as indicated by frequent HCRU and OCS use. This study suggests a high level of remaining unmet need for patients with EGPA, and future studies are needed to understand the impact of new treatments on the patient and disease burden.

## Supplementary material

10.1183/23120541.00430-2023.Supp1 **Please note:** supplementary material is not edited by the Editorial Office, and is uploaded as it has been supplied by the author.
Table S1 00430-2023.table_S1
Supplementary methods and table S2 00430-2023.methods_and_table_S2
Table S3 00430-2023.table_S3
Figure S1 00430-2023.figure_S1
