# Supplementary material for: Prevalence, incidence and healthcare burden of eosinophilic granulomatosis with polyangiitis in the UK
Source: ERJ Open Res. 2024 May 13;10(3):00430-2023. doi: 10.1183/23120541.00430-2023 (PMC11089387; doi:10.1183/23120541.00430-2023)
Supplement: Supplementary file 1 [file 00430-2023.table_S1.pdf]

Supplementary Table S1. *EGPA codes used to identify patients in CPRD-AURUM*

MEDCODE ID	Term	EMIS code	Cleansed READ code	SNOMED CT concept ID
11878901000006118	EGPA	^ESCT1187890		82275008
11878911000006115	EGPA	^ESCT1187891		82275008
3838311000006114	EGPA	^ESCTEO383831		82275008
3838321000006118	Allergic granulomatosis angiitis	^ESCTAL383832		82275008
3838341000006113	Allergic granulomatous angiitis	^ESCTAL383834		82275008
3838351000006110	Churg-Strauss syndrome	^ESCTCS383835		82275008
3838361000006112	Churg Strauss syndrome	^ESCTCH383836		82275008
557151000006115	Churg-Strauss syndrome		G758.00	82275008

EGPA: eosinophilic granulomatosis with polyangiitis; SNOMED: Systematised Nomenclature of Medicine Clinical Terms; ID: identifier.
