# Supplementary material for: Prevalence, incidence and healthcare burden of eosinophilic granulomatosis with polyangiitis in the UK
Source: ERJ Open Res. 2024 May 13;10(3):00430-2023. doi: 10.1183/23120541.00430-2023 (PMC11089387; doi:10.1183/23120541.00430-2023)
Supplement: Supplementary file 2 [file 00430-2023.methods_and_table_S2.pdf]

Supplementary Methods

Sample size/Power calculations

Feasibility assessment of the CPRD-AURUM database identified between 160 and 195 patients with a prevalent EGPA diagnosis recorded during any one year between 2014 and 2018, inclusive. Feasibility assessment of the CPRD-GOLD database identified 4,508,082 patients in 2014 with a minimum one year of data available. Assuming that the CPRD-GOLD database is representative of the UK population, that 84% of the UK population comprises people from England, and that EGPA prevalence in England is the same as that in the UK, CPRD-AURUM comprises an estimated 4.3 – 5.3 million patients with a minimum one year of data available.

The width of the 95% CIs for a range of assumed sample sizes and assumed prevalence and incidence of patients with EGPA were estimated and are shown in **Supplementary Table S2**.

Supplementary Table S2. *95% CIs for (A) prevalence and (B) incidence estimates*

A. *95% CIs for prevalence estimates*

**Assumed sample size (million)**	**Assumed prevalence (per 1,000,000)**	**95% CI (per 1,000,000)**
4	30	(25.0, 36.0)
4	35	(29.5, 41.4)
4	40	(34.1, 46.8)
5	30	(25.5, 35.3)
5	35	(30.1, 40.7)
5	40	(34.7, 46.0)
6	30	(25.8, 34.8)
6	35	(30.5, 40.2)
6	40	(35.2, 45.5)

B. *95% CIs for incidence estimates*

**Assumed sample size (million PY)**	**Assumed incidence (per 1,000,000 PY)**	**95% CI (per 1,000,000 PY)**
4	0.18	(0.00, 1.17)
4	1.20	(0.34, 2.74)
4	4.00	(2.29, 6.50)
5	0.18	(0.00, 0.94)
5	1.20	(0.44, 2.61)
5	4.00	(2.44, 6.18)
6	0.18	(0.00, 0.93)
6	1.20	(0.47, 2.40)
6	4.00	(2.56, 5.95)

CI: confidence interval; PY: person-years.
