# Supplementary material for: Prevalence, incidence and healthcare burden of eosinophilic granulomatosis with polyangiitis in the UK
Source: ERJ Open Res. 2024 May 13;10(3):00430-2023. doi: 10.1183/23120541.00430-2023 (PMC11089387; doi:10.1183/23120541.00430-2023)
Supplement: Supplementary file 3 [file 00430-2023.table_S3.pdf]

Supplementary Table S3. *Prevalence of EGPA in the UK from 2005 to 2019 stratified by age group*

Year	Age group, years					
	All		0–17		≥18	
	EGPA prevalence*	EGPA incidence†	EGPA prevalence*	EGPA incidence†	EGPA prevalence*	EGPA incidence†
2005	22.7 (20.0, 25.7)	N/A	0.51 (0.01, 2.84)	N/A	27.5 (24.2, 31.2)	N/A
2006	25.3 (22.4, 28.4)	3.41 (2.38, 4.74)	0.50 (0.01, 2.79)	0.00	30.7 (27.2, 34.5)	4.14 (2.88, 5.76)
2007	27.7 (24.7, 30.9)	3.64 (2.58, 5.00)	0.49 (0.01, 2.73)	0.00	33.6 (30.0, 37.6)	4.42 (3.13, 6.07)
2008	28.8 (25.8, 32.1)	2.72 (1.82, 3.91)	0.48 (0.01, 2.67)	0.00	35.1 (31.4, 39.1)	3.31 (2.22, 4.75)
2009	30.2 (27.1, 33.5)	2.69 (1.80, 3.86)	0.47 (0.01, 2.61)	0.00	36.8 (33.1, 40.9)	3.27 (2.19, 4.70)
2010	31.8 (28.7, 35.2)	2.74 (1.85, 3.92)	0.46 (0.01, 2.55)	0.00	38.8 (35.0, 42.9)	3.35 (2.26, 4.78)
2011	32.9 (29.7, 36.3)	2.44 (1.61, 3.55)	0.00	0.00	40.4 (36.5, 44.5)	2.98 (1.97, 4.34)
2012	35.6 (32.4, 39.1)	3.46 (2.46, 4.73)	0.00	0.00	43.8 (39.8, 48.2)	4.25 (3.02, 5.81)
2013	38.1 (34.7, 41.7)	3.74 (2.70, 5.06)	0.00	0.00	47.0 (42.9, 51.4)	4.61 (3.33, 6.24)
2014	40.6 (37.1, 44.3)	4.00 (2.91, 5.35)	0.00	0.00	50.4 (46.1, 55.0)	4.95 (3.61, 6.63)
2015	42.2 (38.7, 46.0)	2.96 (2.05, 4.14)	0.00	0.00	52.6 (48.2, 57.2)	3.68 (2.55, 5.14)
2016	42.5 (39.0, 46.2)	2.71 (1.85, 3.83)	0.00	0.00	53.0 (48.6, 57.6)	3.37 (2.31, 4.76)
2017	43.7 (40.2, 47.5)	3.07 (2.16, 4.23)	0.00	0.00	54.5 (50.2, 59.2)	3.82 (2.69, 5.27)
2018	44.9 (41.4, 48.6)	2.68 (1.84, 3.76)	0.00	0.00	56.0 (51.6, 60.7)	3.34 (2.30, 4.68)
2019	45.6 (42.1, 49.4)	2.35 (1.57, 3.38)	0.37 (0.01, 2.09)	0.41 (0.01, 2.30)	56.8 (52.4, 61.4)	2.82 (1.88, 4.08)

*Prevalence expressed as diagnosed EGPA cases per 1,000,000 persons (95% CI); †incidence expressed as newly diagnosed EGPA cases per 1,000,000 person-years (95% CI).

CI: confidence interval; N/A: not applicable.
