# Supplementary figures and images for: Prevalence, incidence and healthcare burden of eosinophilic granulomatosis with polyangiitis in the UK
Source: ERJ Open Res. 2024 May 13;10(3):00430-2023. doi: 10.1183/23120541.00430-2023 (PMC11089387; doi:10.1183/23120541.00430-2023)

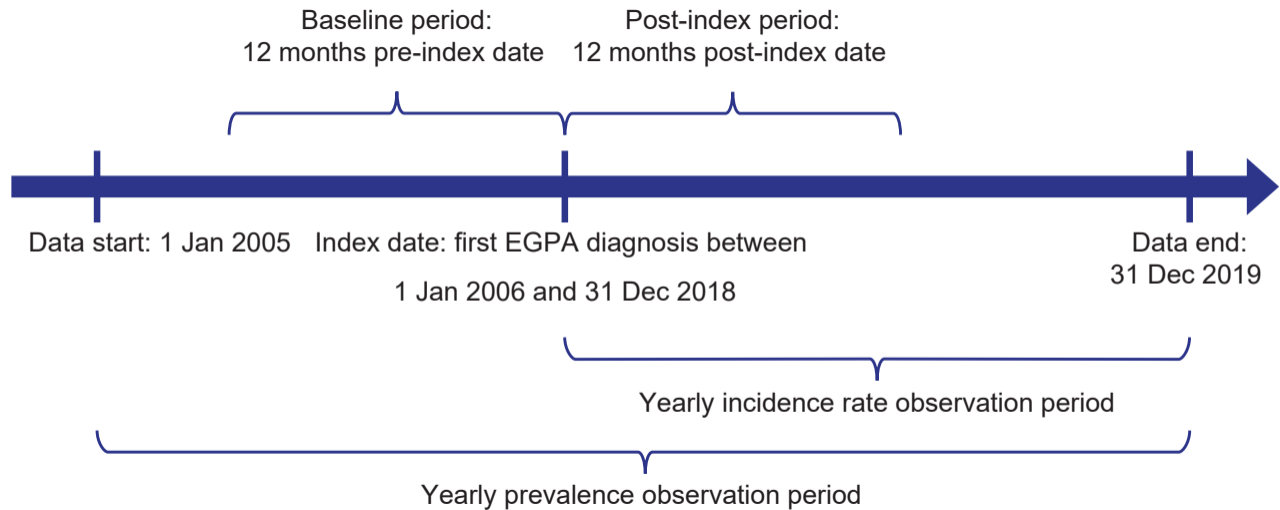

Supplement: Supplementary file 4 [file 00430-2023.figure_S1.pdf]
